# The effect of *CYP2R1* polymorphism (rs10741657) on serum lipid traits in a Han septic population: A case-control study

**DOI:** 10.1097/MD.0000000000040462

**Published:** 2024-11-15

**Authors:** Zhao Lin, Jun Zhou, Siting Wang, Yipan Fan, Xiang Li, Ning Zhang

**Affiliations:** a Department of Intensive Medicine, The Affiliated Jiangning Hospital of Nanjing Medical University, Nanjing, Jiangsu, China.

**Keywords:** *CYP2R1*, lipid, prognosis, sepsis, single nucleotide polymorphism

## Abstract

Vitamin D deficiency has been proven to be associated with dyslipidemia. Additionally, the synthesis of vitamin D depends on cytochrome P450 2R1 (CYP2R1). However, the relationship between *CYP2R1* polymorphisms and lipid metabolism has shown inconsistent results. A case-control study was conducted in a Han Chinese population, including 92 septic patients and 92 polytrauma patients. Based on serum lipid levels, 28 septic patients were further divided into a hyperlipidemia group, while 64 were placed in the control group; similarly, 34 polytrauma patients were categorized into a hyperlipidemia group and 58 into the control group. Genotyping of *CYP2R1*-rs10741657 was performed and serum lipid levels were measured. The Genotype-Tissue Expression project was used to assess expression quantitative trait loci for *CYP2R1* mRNA expression and rs10741657. The genetic analyses revealed that the G-allele of *CYP2R1*-rs10741657 was significantly associated with an increased risk of hyperlipidemia in both sepsis (OR = 2.333, 95% CI: 1.227–4.436, *P* = .010) and polytrauma groups (OR = 4.000, 95% CI: 2.048–7.811, *P* < .001). Further analysis indicated that the rs10741657 mutation was mainly linked to higher serum high-density lipoprotein cholesterol levels in controls (*P* < .05). In functional analysis of rs10741657, the mutation was found to be associated with high *CYP2R1* mRNA expression in whole blood from expression quantitative trait loci data (*P* = 3.53 × 10^−9^). In conclusion, the G-allele of *CYP*2R1-rs10741657 could elevate high-density lipoprotein cholesterol levels and protect against sepsis development.

## 1. Introduction

Sepsis is a life-threatening organ dysfunction caused by a dysregulated host response to infection.^[1]^ The pathophysiological pathways involved in sepsis are complex, including inflammatory signaling, activation of coagulation, and major non-immunological responses.^[2]^ A better understanding of the pathophysiology is beneficial for treating sepsis. Several studies have revealed that serum lipid levels are related to sepsis.^[3–7]^ Therefore, our aim is to explore the role of lipids in sepsis within the Han Chinese population.

The human *CYP2R1* gene is located on chromosome 11p15.2 (gene ID: 120227) and contains 501 amino acids.^[8]^ It encodes 25-hydroxylase, which plays a crucial role in the metabolism of vitamin D.^[9–11]^ Vitamin D is involved in the pathogenesis of many diseases, including autoimmune,^[12]^ infectious,^[13]^ and cardiovascular diseases.^[14]^ Notably, vitamin D has been linked with serum lipid levels.^[6,15,16]^ In European populations, several genome-wide association studies have identified over 25 *CYP2R1* single nucleotide polymorphisms (SNPs) associated with vitamin D deficiency or levels.^[17,18]^ However, it remains worthy to explore whether these SNPs are associated with serum lipid levels.

**Figure 1.**
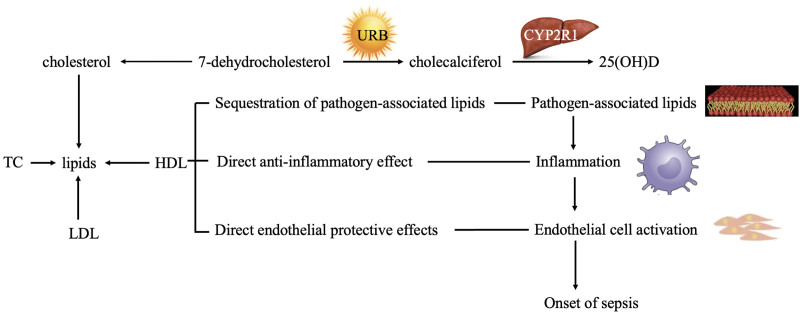
The association between *CYP2R1* along with lipids and sepsis.

Figure 1 illustrates the association between the *CYP2R1* gene, lipids, and sepsis. Therefore, we aim to study the relationship between *CYP2R1* gene polymorphisms and lipid profiles in sepsis in order to provide a theoretical basis for its prevention and treatment.

## 2. Materials and methods

### 2.1. Study population

Sepsis was defined in accordance with the standard criteria.^[1]^ A total of 92 septic patients who were admitted to the Affiliated Jiangning Hospital of Nanjing Medical University from June 2022 to August 2023 were selected as the septic group. During the same period, another 92 cases with polytrauma were selected as the control group. The sample size was determined using the G*Power version (3.1.9.7, http://www.gpower.hhu.de/),^[19]^ the effect size was 0.3, α-level 0.05, β-level 0.9 (power of detection 90%), with one-tailed, based on *t* test family. Hyperlipidemia was defined as a total plasma cholesterol >6.22 mmol/L or low density lipoprotein cholesterol (LDL-C) >4.14 mmol/L or plasma triglycerides (TG) >2.26 mmol/L.^[20]^ All participants with a confirmed diagnosis of hyperlipidemia did not utilize lipid-lowering medications. According to the serum lipid levels, the subjects were further divided into hyperlipidemia group and non-hyperlipidemia. Subjects with a history of cancer, human immunodeficiency disease, immune system disease, hemopathy, chronic disease as well as thyroid dysfunction were excluded from the study. At last, 28 subjects with hyperlipidemia and 64 subjects with non-hyperlipidemia in septic group were admitted; 34 subjects with hyperlipidemia and 58 subjects with non-hyperlipidemia in polytrauma group were admitted.

### 2.2. Clinical measurements

We obtained clinical data, including sex, age, nationality, history of diseases, and smoking history. Blood pressure was measured using a random-zero sphygmomanometer (HM-1101; Hico Medical Co., Ltd., Chiba, Japan) with 20 minutes of rest in a seated position. Body mass index (BMI) was calculated by dividing weight (kg) by height squared (m^2^). The total cholesterol (TC), TG, high-density lipoprotein cholesterol (HDL-C), LDL-C, and fasting blood glucose (GLU) were measured by an automatic biochemical analyzer (Type 7170A; Hitachi Ltd., Tokyo, Japan). The serum levels of 25(OH)D were determined simultaneously using batched specimens with commercially available kits (Eagle Biosciences, Nashua, NH) and were conducted in accordance with the manufacturer’s instructions in prior studies, which confirmed the validity of the test. Peripheral blood collection was performed as soon as the patient was enrolled in the study, within the first 72 hours of sepsis diagnosis.

### 2.3. Genotyping

Genomic DNA was extracted from the peripheral blood leukocytes from every subject using the Tiangen Blood DNA Kit (TIANamp Blood DNA Kit; Tiangen Biotech, Beijing, China). The *CYP2R1*-rs10741657 was genotyped using TaqMan Genotype assays (catalog no. 4351379 C_2958430_10, Thermo Fisher Scientific, USA) according to the supplier’s recommendations. The allele discrimination was clearly observed for each TaqMan assay. Duplication of genotyping for approximately 5% of samples was applied for quality control.

### 2.4. Expression quantitative trait loci (eQTL)

The publicly available RNA-seq and 670 genotyping data of whole blood were provided by Genotype-Tissue Expression project (GTEx Portal; https://www.gtexportal.org/home), which were applied to assess eQTL for *CYP2R1* gene mRNA expression and rs10741657.^[21]^

### 2.5. Protein–protein interaction (PPI) networks

The STRING database (https://string-db.org/) was used to construct the PPI networks to identify the interactive relationships between CYP2R1, CYP24A1, CYP27B1, and CYP27A1.

### 2.6. Statistical analyses

All data were analyzed by using the SPSS software, version 22.0 (SPSS, Chicago, IL). Variables are displayed as a percentage or the mean ± SD. Differences between groups were calculated by the χ^2^ test or Student *t* test. The Hardy–Weinberg equilibrium was assessed for controls by use of the χ^2^ test. The associations between the *CYP2R1*-rs10741657 and hyperlipidemia were tested by logistic regression analysis. The associations between rs10741657 and serum lipids levels were calculated by multivariate linear regression. All of the models used in the study were adjusted for age, sex, BMI, smoking, systolic blood pressure (SBP), diastolic blood pressure (DBP), and GLU unless otherwise noted.

## 3. Results

### 3.1. Associations between sepsis and serum lipids levels

**Figure 2.**
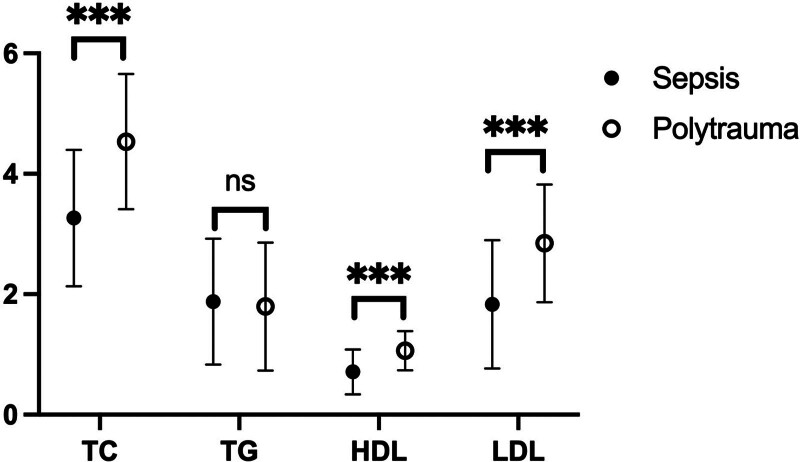
The association between sepsis and the serum lipids levels. ***P < .001; ns, *P* > .05.

The associations between sepsis and serum lipid levels are summarized in Figure 2. The serum levels of total TC, HDL-C, and LDL-C were significantly lower in the septic group compared to the polytrauma group (all, *P* < .001). However, the TG levels were similar between the septic and polytrauma groups (*P* > .05). Furthermore, stratifying analysis was performed on 2 subgroups: hyperlipidemia groups and controls.

### 3.2. Demographic characteristics of hyperlipidemia patients and controls

The clinical characteristics of hyperlipidemia patients and controls in both septic and polytrauma groups are summarized in Table 1. Hyperlipidemia patients and controls were matched for age and sex in both groups.

**Table 1:** Comparison of clinical characteristics between hyperlipidemia and control.

	Sepsis (N = 92)			Polytrauma (N = 92)		
	Hyperlipidemia (N = 28)	Control (N = 64)	*P* value	Hyperlipidemia (N = 34)	Control (N = 58)	*P* value
Age (yr)	69.39 ± 14.42	72.88 ± 12.12	.235	62.35 ± 14.28	64.26 ± 15.87	.566
Males (%)	17 (60.71)	41 (64.06)	.759	18 (52.94)	33 (56.90)	.713
Smoking (%)	4 (14.29)	11 (17.19)	.729	5 (14.71)	4 (6.90)	.224
SBP (mm Hg)	92.93 ± 22.71	89.66 ± 25.04	.555	138.74 ± 36.50	137.62 ± 24.67	.862
DBP (mm Hg)	54.00 ± 13.94	53.77 ± 14.05	.941	85.56 ± 25.54	78.81 ± 13.17	.098
BMI (kg/m^2^)	22.80 ± 3.19	23.51 ± 3.29	.337	23.81 ± 3.81	22.84 ± 4.07	.263
TC (mmol/L)	3.52 ± 1.21	3.16 ± 1.09	.162	5.26 ± 1.22	4.11 ± 0.81	<.001
TG (mmol/L)	3.12 ± 0.97	1.34 ± 0.44	<.001	2.81 ± 1.08	1.21 ± 0.41	<.001
HDL-C (mmol/L)	0.78 ± 0.32	0.66 ± 0.38	.155	1.07 ± 0.30	1.06 ± 0.34	.886
LDL-C (mmol/L)	2.21 ± 1.30	1.67 ± 0.90	.023	3.55 ± 0.99	2.43 ± 0.70	<.001
GLU (mmol/L)	7.81 ± 2.94	10.84 ± 4.96	.003	6.30 ± 2.97	6.38 ± 2.62	.896
25(OH)D (nmol/L)	49.08 ± 9.33	48.80 ± 8.82	.890	56.55 ± 7.29	54.25 ± 10.90	.277

BMI = body mass index, DBP = diastolic blood pressure, GLU = fasting blood glucose, HDL-C = high-density lipoprotein cholesterol, LDL-C = low-density lipoprotein cholesterol, SBP = systolic blood pressure, TC = total cholesterol, TG = triglycerides.

In the septic group, there were no significant differences in SBP, DBP, BMI, as well as the serum levels of TC, HDL-C, and 25(OH)D between hyperlipidemia patients and controls (all, *P* > .05). However, the levels of TG and LDL-C were higher in hyperlipidemia patients than in controls (all, *P* < .05). Additionally, the levels of GLU were lower in hyperlipidemia patients than controls (*P* < .05).

In the polytrauma group, there were no differences in SBP, DBP, and BMI along with the serum levels of HDL-C, GLU, and 25(OH)D between hyperlipidemia patients and controls (all, *P* > .05). However, the levels of TC, TG, and LDL-C were higher in hyperlipidemia patients than controls (all, *P* < .05).

### 3.3. Associations between *CYP2R1*-rs10741657 and hyperlipidemia

The associations between *CYP2R1*-rs10741657 and hyperlipidemia in septic and polytrauma groups are summarized in Table 2. The genotype distributions for SNP complied with Hardy–Weinberg equilibrium values in the controls.

**Table 2:** Association between *CYP2R1*-rs10741657 and hyperlipidemia.

Stratum	Group	AA	AG + GG	Genotype OR (95% CI)*			Allele*		
				Additive	Dominant	Recessive	A/G	OR (95% CI)	*P* value
Sepsis	Hyperlipidemia	6	10 + 12	1.536 (0.593–3.977) *P* = .377	3.235 (1.158–9.040) *P* = .025	2.396 (0.948–6.055) *P* = .065	22/34	2.333 (1.227–4.436)	.010
	Control	30	17 + 17				77/51		
Polytrauma	Hyperlipidemia	4	8 + 22	1.923 (0.648–5.711) *P* = .239	7.000 (2.187–22.407) *P* = .001	3.000 (1.243–7.239) *P* = .015	16/52	4.000 (2.048–7.811)	<.001
	Control	28	8 + 22				64/52		

The A-allele was used as the reference. Additive model: AG vs. GG + AA; dominant model: GG + AG vs. AA; and recessive model: GG vs. AG + AA.

BMI = body mass index, DBP = diastolic blood pressure, GLU = fasting blood glucose, SBP = systolic blood pressure.

* Adjusted for age, sex, BMI, smoking, SBP, DBP, and GLU.

In the septic group, the rs10741657 G-allele was strongly associated with an increased risk for hyperlipidemia adjusted for age, sex, BMI, smoking, SBP, DBP, and GLU (OR = 2.333, 95% CI: 1.227–4.436, *P* = .010). rs10741657 was also associated with hyperlipidemia in the dominant model (OR = 3.235, 95% CI: 1.158–9.040, *P* = .025). No significant association was found in the additive (OR = 1.536, 95% CI: 0.593–3.977, *P* = .377) and recessive (OR = 2.396, 95% CI: 0.948–6.055, *P* = .065) models.

In the polytrauma group, a significant association was found between the rs10741657 G-allele and an increased risk for hyperlipidemia adjusted for age, sex, BMI, smoking, SBP, DBP, and GLU (OR = 4.000, 95% CI: 2.048–7.811, *P* < .001). rs10741657 was also associated with hyperlipidemia in the dominant (OR = 7.000, 95% CI: 2.187–22.407, *P* = .001) and recessive (OR = 3.000, 95% CI: 1.243–7.239, *P* = .015) models; however, no significant association was found in the additive model (OR = 1.923, 95% CI: 0.648–5.711, *P* = .239).

### 3.4. Associations between *CYP2R1*-rs10741657 and serum lipids levels

The associations between *CYP2R1*-rs10741657 and serum lipid levels in septic and polytrauma groups are summarized in Table 3. In the septic group, the rs10741657 G-allele was significantly associated with higher HDL-C levels in both controls of septic group (*P* = .049) and polytrauma group (*P* = .018). Furthermore, in the polytrauma group, the rs10741657 G-allele was also related to higher TG levels in controls (*P* = .002). No other significant association was found between rs10741657 polymorphism and serum lipid levels (all, *P* > .05).

**Table 3:** Quantitative trait analysis for serum lipid levels between genotypes of *CYP2R1*-rs10741657.

Stratum	Genotype	TC (mmol/L)		TG (mmol/L)		HDL-C (mmol/L)		LDL-C (mmol/L)	
		Hyperlipidemia	Control	Hyperlipidemia	Control	Hyperlipidemia	Control	Hyperlipidemia	Control
Sepsis	AA	3.41 ± 0.94 (N = 6)	2.98 ± 1.15 (N = 30)	3.41 ± 1.22 (N = 6)	1.23 ± 0.43 (N = 30)	0.70 ± 0.36 (N = 6)	0.57 ± 0.26 (N = 30)	2.00 ± 0.84 (N = 6)	1.58 ± 0.99 (N = 30)
	AG	3.41 ± 1.48 (N = 10)	3.15 ± 0.81 (N = 17)	2.75 ± 0.67 (N = 10)	1.45 ± 0.40 (N = 17)	0.75 ± 0.24 (N = 10)	0.65 ± 0.38 (N = 17)	1.82 ± 1.43 (N = 10)	1.97 ± 0.64 (N = 17)
	GG	3.66 ± 1.16 (N = 12)	3.48 ± 1.21 (N = 17)	3.29 ± 1.04 (N = 12)	1.42 ± 0.47 (N = 17)	0.85 ± 0.37 (N = 12)	0.85 ± 0.50 (N = 17)	2.64 ± 1.34 (N = 12)	1.51 ± 0.95 (N = 17)
	F	0.144	1.172	1.186	1.884	0.496	3.169	1.219	1.363
	P*	0.866	0.317	0.322	0.161	0.615	0.049	0.312	0.264
Polytrauma	AA	5.72 ± 1.72 (N = 4)	3.98 ± 0.97 (N = 28)	2.15 ± 1.39 (N = 4)	1.03 ± 0.34 (N = 28)	0.88 ± 0.16 (N = 4)	0.95 ± 0.28 (N = 28)	3.99 ± 1.83 (N = 4)	2.31 ± 0.87 (N = 28)
	AG	4.85 ± 1.20 (N = 8)	4.36 ± 0.42 (N = 8)	2.87 ± 1.37 (N = 8)	1.24 ± 0.46 (N = 8)	1.06 ± 0.36 (N = 8)	1.01 ± 0.17 (N = 8)	3.11 ± 0.83 (N = 8)	2.59 ± 0.51 (N = 8)
	GG	5.33 ± 1.15 (N = 22)	4.18 ± 0.67 (N = 22)	2.90 ± 0.91 (N = 22)	1.42 ± 0.38 (N = 22)	1.11 ± 0.30 (N = 22)	1.22 ± 0.41 (N = 22)	3.64 ± 0.84 (N = 22)	2.52 ± 0.47 (N = 22)
	F	0.767	0.817	0.817	6.994	0.996	4.297	1.320	0.770
	P*	0.473	0.447	0.451	0.002	0.392	0.018	0.282	0.468

BMI = body mass index, DBP = diastolic blood pressure, GLU = fasting blood glucose, HDL-C = high-density lipoprotein cholesterol, LDL-C = low-density lipoprotein cholesterol, TC = total cholesterol, TG = triglycerides, SBP = systolic blood pressure.

* Adjusted for age, sex, BMI, smoking, SBP, DBP, and GLU.

### 3.5. The function analysis of *CYP2R1*-rs10741657

**Figure 3.**
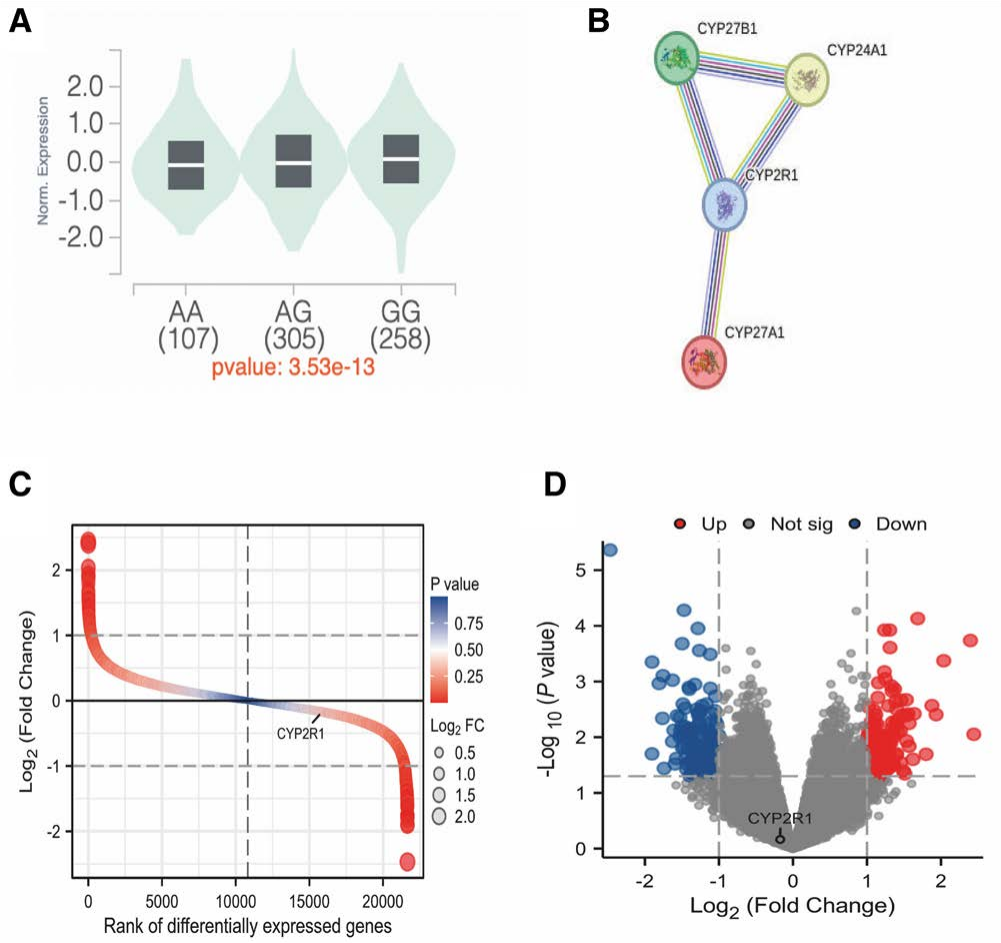
The functional analysis of *CYP2R1*. (A) The association between *CYP2R1*-rs10741657 and *CYP2R1* mRNA expression through eQTLs analysis. (B) Protein-protein interaction network constituted with CYP2R1, CYP24A1, CYP27B1, and CYP27A1. (C) Difference ranking plot of DEGs between the obese subjects and control samples screened from GSE22435 database. (D) Volcano plot of DEGs between the obese subjects and control samples screened from GSE22435 database. DEGs = differentially expressed genes.

The eQTLs for rs10741657 and *CYP2R1* mRNA expression were provided by the Genotype-Tissue Expression project (Fig. 3A). The eQTL data demonstrated that mutation at rs10741657 is associated with higher *CYP2R1* mRNA expression in whole blood (*P* = 3.53 × 10^−9^).

Our analysis yielded the following network stats: an average node degree of 2, an average local clustering coefficient of 0.833, and a PPI enrichment *P*-value of 2.58 × 10^−8^ (Fig. 3B). These metrics suggest a significant level of interconnectivity and a nonrandom pattern of interaction among these biomarkers, supporting the hypothesis that they are indeed closely associated within the cellular pathways affected by lipid metabolism.

## 4. Discussion

The study explored the associations among *CYP2R1* polymorphisms, lipid concentrations, and sepsis. The results showed a significant association between sepsis and serum TC, HDL-C, and LDL-C levels in the Han Chinese population. Subgroup analysis was performed on hyperlipidemia groups and controls. The G-allele of *CYP2R1*-rs10741657 was found to be associated with an increased risk of hyperlipidemia and higher levels of HDL-C in controls. eQTL and PPI analysis also confirmed the association between *CYP2R1* and lipids. Therefore, the rs10741657 G-allele could elevate HDL-C levels and protect against sepsis development.

Sepsis is a systemic inflammatory disease with high morbidity and mortality caused by a dysregulated host immune response to infection.^[1]^ The reasons for sepsis-elicited organ injury and mortality are complex interactions between microbes, blood cells, and endothelium resulting in multi-organ microcirculatory failure.^[22]^ A better understanding of the pathophysiology is beneficial for treating sepsis.

Serum lipid levels play an important role in pro-inflammatory changes as well as counterregulatory anti-inflammatory changes in the microvasculature during sepsis.^[4]^ Previous studies have shown that low LDL-C levels were related to higher long-term rates of sepsis,^[23]^ while patients with culture-positive bacteremia of sepsis had lower cholesterol levels.^[24]^ In sepsis patients, a U-shaped curve was demonstrated between non-HDLc/HDLc ratio and 28-day mortality risk.^[25]^ These findings are consistent with our study’s indication of a significant association between lower serum TC, HDL-C, as well as LDL-C levels with sepsis.

The correlation between cholesterol metabolism and vitamin D is evident. A common metabolic substrate, 7-DHC, is shared between the two. In reality, 7-DHC is converted to vitamin D in the skin upon sun exposure; however, it can also be converted to cholesterol by 7-DHCR. It is possible that polymorphisms associated with vitamin D may have a relationship with lipid metabolism. In our study, we found that the rs10741657 G-allele in *CYP2R1* was associated with an increased risk of hyperlipidemia and higher levels of HDL-C in controls. Similarly, in a Lebanese cohort, the rs10741657 G>A variant in *CYP2R1* was linked to circulating LDL-C and HDL-C levels.^[26]^ A cross-sectional population-based study involving 460 unrelated Lebanese individuals revealed that SNPs (rs4986790 and rs4986791) in toll-like receptor 4 were negatively correlated with serum vitamin D levels, which in turn were inversely associated with *H pylori* infection. Furthermore, individuals infected with *H pylori* exhibited elevated serum TC levels.^[27]^ These findings were consistent; however, data from differentially expressed genes showed no significant association between *CYP2R1* and obesity (which is related to lipids) (Fig. 3C and D). The discrepancies may be attributed to differences in race and sample size.

The associations among *CYP2R1*, lipids, and sepsis are outlined in Figure 1. Specifically, *CYP2R1*-rs10741657 was associated with serum HDL levels; which are known to be related to sepsis development.^[7]^ The role of HDL in sepsis was summarized as well. It could isolate and neutralize pathogen-associated lipids, exert direct anti-inflammatory effects, and counteract activation of the endothelium caused by cytokines.^[28]^ Additionally, HDL could upregulate activating transcription factor 3,^[29]^ a negative regulator in the toll-like receptor 4 signaling pathway,^[30]^ which plays a role in negatively regulating NF-κB via direct interaction with p65.^[31]^

In conclusion, the rs10741657 G-allele in *CYP2R1* was found to be associated with increased levels of HDL-C as well as an elevated risk of hyperlipidemia within the Han Chinese population. Furthermore, sepsis was found to be correlated with TC, HDL-C, and LDL-C concentrations. Therefore, *CYP2R1*-rs10741657 G-allele might elevate HDL-C levels and protect against sepsis development.

These findings need confirmation on a larger scale. Further investigations are needed to explore the associations among vitamin polymorphisms, lipids, and sepsis. A better understanding of this relationship may reduce the risk of developing sepsis and provide a new treatment strategy.

## Author contributions

**Data curation:** Jun Zhou.

**Formal analysis:** Yipan Fan.

**Funding acquisition:** Zhao Lin, Ning Zhang.

**Investigation:** Zhao Lin, Jun Zhou.

**Methodology:** Siting Wang.

**Software:** Siting Wang, Xiang Li.

**Validation:** Yipan Fan.

**Visualization:** Xiang Li.

**Writing – original draft:** Zhao Lin.

**Writing – review & editing:** Ning Zhang.
